# The Virtues and Vices of Pfs230: From Vaccine Concept to Vaccine Candidate

**DOI:** 10.4269/ajtmh.21-1337

**Published:** 2022-07-11

**Authors:** Patrick E. Duffy

**Affiliations:** Laboratory of Malaria Immunology and Vaccinology, National Institute of Allergy and Infectious Diseases, National Institutes of Health, Bethesda, Maryland

## Abstract

Among the *Plasmodium falciparum* surface antigens reported by Richard Carter and his colleagues decades ago, Pfs230 is currently the target of the most advanced candidate for a malaria transmission-blocking vaccine. First identified by its orthologue in the avian malaria parasite *Plasmodium gallinaceum*, the large cysteine-rich 14-domain Pfs230 antigen is displayed on the surface of gametes that emerge in the mosquito midgut. Gametes lacking Pfs230 cannot bind to red blood cells nor develop further into oocysts. Human antibodies against Pfs230 lyse gametes in the presence of complement, which largely explains serum transmission-blocking activity in Pfs230 antisera. A protein–protein conjugate vaccine that incorporates the first domain of the Pfs230 antigen induced greater serum transmission-reducing activity versus a similarly manufactured Pfs25 vaccine in U.S. trials, and is currently in phase II field trials in Mali.

The gamete surface antigen Pfs230 is the target of the leading candidate for a malaria transmission-blocking vaccine (TBV). The full-length 360-kDa Pfs230 precursor is expressed by gametocytes within erythrocytes and is processed to become an approximately 300-kDa mature protein upon translocation to the surface of gametes that emerge in the mosquito midgut.^1^ Gametes lacking Pfs230 cannot bind to red blood cells nor develop further into oocysts.^2^ Pfs230 is displayed on the surface of gametes and zygotes in a complex with the integral membrane antigen Pfs48/45, then sheds as zygotes transition to ookinetes.^3^

Antibodies against Pfs230 can exert their effects before gamete fertilization; hence, Pfs230 is referred to as a pre-fertilization target.^4^ Because Pfs230 induces antibodies during human infection,^5^ naturally occurring infections might boost vaccine responses to Pfs230 and prolong vaccine durability. Pfs230 antibodies lyse *Plasmodium falciparum* gametes in the presence of complement,^6^ which enhances serum activity at a given antibody titer and thus may also have the effect of prolonging vaccine activity. The initial six-cysteine domain of the 14-domain Pfs230 antigen has been incorporated into a protein–protein conjugate vaccine that is the first candidate to enter the clinic and is currently being assessed in phase II field trials in Mali.

The discovery and initial characterization of Pfs230 parallel in many ways the Pfs25 story.^7^ Following seminal studies that established the concept of transmission-blocking immunity,^8^^,^^9^ Richard Carter set out to identify the *Plasmodium* sexual-stage surface antigens targeted by transmission-blocking antibodies. These studies at the Laboratory of Parasitic Diseases at the National Institute of Allergy and Infectious Diseases began with surface radioiodination of *Plasmodium gallinaceum* gametes using the robust system for generating sexual-stage parasites previously established by Carter and Chen.^8^ Among several *P. gallinaceum* surface antigens identified by this approach, surface radioiodination labeled a 240-kD protein on gametes that was lost from the surface over the 12 to 24 hours of development after induction of gametogenesis.^10^ This 240-kD antigen appeared as part of a complex immunoprecipitated with complement-dependent transmission-blocking monoclonal antibodies (mAbs).^11^ Subsequently, Carter and his colleagues at the Laboratory of Parasitic Diseases demonstrated that Pfs230, the *P. falciparum* equivalent of the *P. gallinaceum* 240-kD gamete surface antigen, was itself a target of complement-dependent transmission-blocking mAbs.^4^

Thus was born a contestant in one of the malaria “vaccine races,” as Carter would call them—in this case, the race to develop a malaria TBV. However, Pfs230 has covered the course in fits and starts. The next lap for Pfs230 was completed by David Kaslow, who succeeded Carter to lead sexual-stage malaria research at NIH, together with his postdoctoral fellow Kim Williamson. Williamson and Kaslow were the first to report the sequence of Pfs230,^1^ using the approach that Kaslow pioneered for Pfs25: parasite antigen was immunoprecipitated with functional mAbs, tryptic peptides sequenced to design degenerate nucleotide probes, and complementary DNA fragments cloned from a library with the probes and then sequenced. With the knowledge that Pfs230 shared a cysteine motif with the malaria surface antigen Pf12,^1^ Carter took the baton for the next lap; he surmised that Pfs230 comprised 14 domains with an even number of cysteines (two, four, or six) that formed disulfide bonds within each domain (Figure 1).^12^ This structure prediction was subsequently confirmed by comparative modeling with the *Toxoplasma gondii* antigen SAG1,^13^ and ultimately by the crystal structure of recombinant Pfs230 domain 1.^14^^,^^15^ These domains are referred to as six-cysteine (6-Cys) domains and are shared among a family of *Plasmodium* proteins, including the TBV target antigens Pfs48/45 and Pfs47 (see contributions by R. Sauerwein and by C. Barillas-Mury in this issue). Carter’s domain architecture provided the blueprint that scientists have since used to design subunit Pfs230 immunogens.

**Figure 1.**
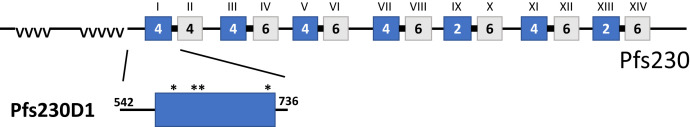
Schematic of native Pfs230 and the Pfs230D1 domain incorporated in the first Pfs230 vaccine to enter clinical trials. Roman numerals show 14 disulfide bond-containing domains, Arabic numerals within boxes indicate the number of cysteines in each domain, and the paired blue and gray boxes represent the seven double domains of Pfs230 defined by Gerloff et al.^12^ Asterisks denote the position of cysteines within Pfs230D1; Arabic numerals indicate construct boundaries for the Pfs230D1 vaccine antigen (amino acids 542–736 of the Pfs230 NF54 allele).

Williamson and Kaslow first showed that a recombinant Pfs230 immunogen can induce transmission-blocking antibodies, using an N-terminal Pfs230 fragment (amino acid [aa] 443–1,132) encompassing 6-Cys domains 1 and 2, prepared as a fusion with maltose-binding protein, to immunize mice.^16^^,^^17^ Subsequent preclinical studies similarly showed that recombinant Pfs230 fragments incorporating the first disulfide-bonded domain (domain 1, aa 589–730 in the NF54 allele^12^) can induce serum transmission-blocking activity in animals,^18^^–^^21^ as can the N-terminal peptide upstream of domain 1.^22^^,^^23^ Williamson was also first to show that chemical conjugation of a recombinant Pfs230 immunogen to a protein carrier—in this case, tetanus toxoid—could enhance immunogenicity in mice.^24^

These findings positioned the field to advance a Pfs230 candidate to the clinic. However, numerous factors hindered progress: the large size of the full-length Pfs230 antigen; difficulty in expressing 6-Cys proteins, including Pfs230 domains, as properly folded antigens; the dearth of resources to advance malaria vaccines in general (and even more so for TBV); and the focus on Pfs25 and its *Plasmodium vivax* orthologue Pvs25 as the leading TBV candidates. Preclinical studies supported the emphasis on Pfs25 candidates. In rodents, Pfs25 immunogens appeared as potent or more potent than other TBV immunogens, including Pfs230.^25^^,^^26^ Carter himself had concerns for major histocompatibility complex restriction of responses to Pfs230 based on early studies,^27^^,^^28^ although his subsequent work in malaria-experienced populations did not support this idea.^29^ Instead, it was suggested that immunodominance of the glutamate-rich N-terminal fragment (which is cleaved off during gametogenesis^30^) impaired responses to the downstream elements in the processed Pfs230 protein.^31^

Clinical development of a Pfs230 vaccine candidate began in earnest at the Laboratory of Malaria Immunology and Vaccinology (LMIV), National Institute of Allergy and Infectious Diseases, shortly after its inauguration in 2009 (Figure 2). The LMIV clinical development plan envisioned advancing a Pfs25 candidate to the clinic followed by a Pfs230 candidate for studies that compared and combined their activity. Using a quality-by-design strategy, the LMIV developed and manufactured a recombinant Pfs230 domain 1 (Pfs230D1) antigen corresponding to aa sequence positions 542 to 736 of the full-length Pfs230 (NF54 allele), with *Pichia pastoris* as the production system.^32^ To enhance immunogenicity, LMIV investigators chemically conjugated Pfs230D1^33^ (as well as Pfs25^34^) to ExoProtein A (EPA), a recombinant mutant and detoxified exotoxin from *Pseudomonas aeruginosa*. EPA is not a component of any licensed vaccine, but has been studied extensively as a component of conjugated typhoid and shigellosis vaccines.^35^^–^^37^

**Figure 2.**
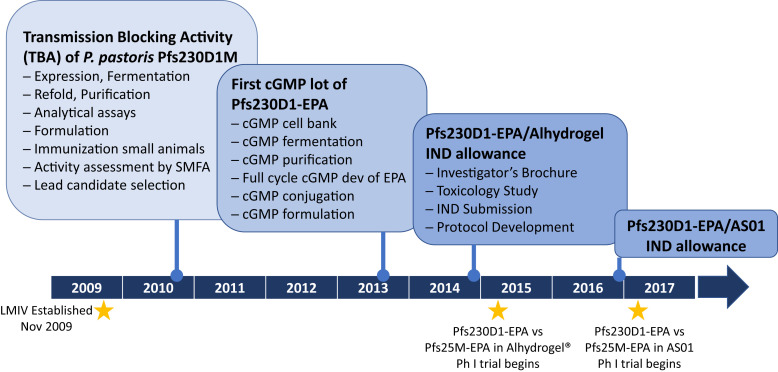
Major milestones in the development of the first Pfs230 candidate, Pfs230D1–ExoProtein A (EPA), to enter clinical trials. In 2010, the Laboratory of Malaria Immunology and Vaccinology (LMIV) at NIAID, NIH, committed to cGMP manufacture and clinical trials of the protein–protein conjugate vaccine Pfs230D1-EPA formulated in Alhydrogel^®^. The first cGMP lot was available in 2013 and, after preclinical toxicology testing and protocol development, an Investigational New Drug (IND) application was reviewed and allowed by the U.S. Food and Drug Administration in late 2014. The first human study of Pfs230D1-EPA formulated in Alhydrogel started at the NIH Clinical Center in Bethesda, Maryland, in 2015, and the first human study of Pfs230D1-EPA formulated in the GSK adjuvant AS01 started in 2017 at the Sotuba Clinical Research Center in Bamako, Mali, managed by the Malaria Research and Training Center of the University of Sciences, Techniques and Technologies of Bamako. Ph = phase; SMFA = standard membrane-feeding assay.

In the first field trial of any TBV, the LMIV partnered with scientists from the Malaria Research and Training Center at the University of Bamako led by Ogobara Doumbo to assess the lead Pfs25 candidate developed at the LMIV: Pfs25M-EPA formulated in Alhydrogel^®^ (Brenntag, Frederikssund, Denmark). Although the teams found that Pfs25M-EPA/Alhydrogel induced serum functional activity in Malian adults measured in vitro by standard membrane-feeding assay, antibody titers dropped rapidly after their peak,^38^ similar to results in U.S. vaccinees in whom vaccine activity was brief.^39^

The LMIV then examined the hypothesis that the Pfs230D1-EPA candidate, alone or in combination with Pfs25M-EPA, would improve TBV functional activity when both were formulated in Alhydrogel. As seen in prior small-animal studies, the two candidates induced similar serum functional activity in mice.^40^ However, Pfs230D1-EPA induced significantly greater activity than Pfs25M-EPA in rhesus monkeys when tested in the presence of complement. In a trial in malaria-naive U.S. adults, four of five Pfs230D1-EPA/Alhydrogel recipients developed substantial serum functional activity that depended on complement after only two doses; none of the five Pfs25-EPA recipients developed significant activity after two doses. Furthermore, Pfs230D1-EPA co-administered with Pfs25-EPA did not increase activity over Pfs230D1-EPA alone. Thus, the complement-dependent functional immunogenicity of Pfs230D1 represented a significant improvement over Pfs25; monkeys, but not mice, predicted that Pfs230D1 would be superior to Pfs25 in humans.^40^

Trials in Mali are now examining the Pfs230D1-EPA conjugate vaccine candidate in malaria-experienced populations (clinical trials NCT02942277 and NCT03917654). These studies are evaluating Pfs230D1-EPA alone or in combination with Pfs25-EPA. In addition, the Mali trials are examining different adjuvants as another strategy to maximize functional immunogenicity (Figure 2).

The molecular basis for Pfs230D1 vaccine activity in humans has been examined. Human mAbs to Pfs230D1 were generated, using B cell receptor sequences of Pfs230D1-reactive B cells sorted from Malian adults after Pfs230D1-EPA/Alhydrogel vaccination.^14^ Among nine recombinant IgG1 mAbs examined, only one (LMIV230-01) manifested high transmission-blocking activity, and mAb activity largely corresponded to reactivity to native antigen on the gamete surface. LMIV230-01 lysed gametes and blocked oocyst formation in mosquitoes, but these functional activities were reduced substantially in heat-inactivated sera, confirming that complement enhances activity of Pfs230D1-induced antibodies. In addition, LMIV230-01 induced complement membrane attack complex to form on the surface of live *P. falciparum* gametes in the presence of intact, but not heat-inactivated, serum.^14^ Notably, some transmission-reducing activity measured in vitro can be retained after heat inactivation of human Pfs230 antisera, and this phenomenon is more prominent in monkey Pfs230 antisera.^40^ However, the contribution of gamete lysis without complement versus other mechanisms of gamete neutralization to serum activity is unknown and requires further study.

In structural analyses, all six complementarity-determining regions of the LMIV230-01 mAbs were seen to contact Pfs230D1, forming an extensive epitope surface area (1,047 Å^2^). The discontinuous conformational epitope on Pfs230D1 was spread across five β-strands and an N-terminal loop within Pfs230D1. Importantly, the LMIV230-01 epitope appears to be highly conserved across a database of African and Asian *P. falciparum* genomes. Five polymorphisms in or near the epitope did not reduce binding affinity in mutational studies.^14^

The molecular antibody data demonstrate that Pfs230D1 vaccination can induce potent transmission-blocking antibodies in humans that bind native antigen on gametocytes, gametes, and zygotes, and reduce gamete fertilization in the mosquito vector. However, Pfs230D1vaccine recipients generate both functional and non-functional antibodies, and they specifically vary in their levels of antibody that target the LMIV230-01 epitope. The identification of a large, potent transmission-blocking epitope provides a basis to improve the design of Pfs230D1-based TBVs, for example by focusing the antibody response on the LMIV230-01 epitope and removing non-functional epitopes.

Although Pfs25 immunogen design may be improved similarly through structural vaccinology,^41^ attention is focused increasingly on Pfs230 immunogens. Additional expression platforms are being explored for recombinant production of N-terminal Pfs230 fragments with varying domain boundaries, including *Nicotiana benthamiana*,^42^ the baculovirus system,^19^^,^^20^^,^^43^ *Hansenula polymorpha*,^44^^,^^45^ and *Lactococcus lactis*.^20^ New Pfs230 candidates have been prepared as nanoparticle immunogens through incorporation in virus-like particles,^44^^,^^45^ conjugation to bacterial membrane vesicles (outer membrane protein complex derived from *Neisseria meningitidis*),^46^ or display on the surface of liposomes,^47^^,^^48^ and all these approaches have induced serum functional activity in preclinical studies. New vaccine platforms, particularly adjuvants, can enhance the level and duration of antibody responses to malaria antigens, and different adjuvants are already being tested with Pfs230 vaccine candidates (clinical trials NCT05135273).

Richard Carter was a champion of Pfs230 as a target of TBVs because of the striking complement-mediated lytic effects of Pfs230 antibodies^6^ and the evidence that these are induced during natural infections.^5^ Ongoing field trials of Pfs230 candidates can assess the benefit of natural boosting to enhance or sustain vaccine responses. In the updated version of his two-part *Studies with Malaria: A Memoir*,^49^ Carter concluded with a chapter on “an ideal malaria transmission blocking vaccine”, enthusing: “There is one, and only one [emphasis in original], malaria gamete surface antigen that absolutely, and invariably, fits this bill. It is, in the specific case of *Plasmodium falciparum*, Pfs230” (p. 297). The malaria vaccine community may be coming around to his thinking, and perhaps the technologies required to generate effective Pfs230-based vaccines are getting there as well. If Pfs230 has at times been a tortoise in the vaccine races, tortoises have virtues and admirers; Pfs230 may yet plod across the finish line ahead of the field.
